# Subdural hematoma after cesarean delivery without symptoms: a case report

**DOI:** 10.1186/s40981-018-0151-8

**Published:** 2018-02-20

**Authors:** Syuhei Domoto, Manzo Suzuki, Shinpei Suzuki, Hiroyasu Bito

**Affiliations:** Department of Anesthesiology, Musashikosugi Hospital, Nippon Medical School, 1-396 Kosugi-cho, Nakahara-ku, Kawasaki, Kanagawa 211-8533 Japan

**Keywords:** Neuraxial anesthesia, Subdural hematoma, Symptom-free

## Abstract

**Background:**

Subdural hematoma (SDH) after accidental dural puncture (ADP) is rare but may be lethal. We experienced a patient who developed SDH after combined spinal and epidural anesthesia without a headache.

**Case presentation:**

A 41-year-old parturient female with an unruptured cerebral aneurysm, was scheduled to undergo elective cesarean delivery. Cerebrospinal fluid leakage was identified during puncture of the epidural space, and a catheter was placed after re-puncture. The postoperative course was normal except for incisional pain. The patient complained of slight neck stiffness on the second postoperative day. Magnetic resonance imaging (MRI) was performed to ensure that there was no intracranial problem on the fourth postoperative day and showed a bilateral subdural hematoma. Increase in size of hematoma was shown on computed tomography (CT) on the ninth postoperative day. Epidural blood patch was performed. A decrease in the size of the hematoma was confirmed on the CT images on the 11th postoperative day, and the patient was discharged. The patient has not developed any additional symptoms.

**Conclusions:**

We experienced a patient who developed a SDH without a remarkable headache. It may be better to perform brain imaging studies, even if the patient does not complain of headache.

## Background

Chronic subdural hematoma (CSDH) following accidental dural puncture (ADP) is a rare complication after neuraxial anesthesia. Many cases of CSDH after ADP have occurred in patients after spinal anesthesia [1–4]. Cases of CSDH after epidural anesthesia have also been reported [5, 6]. The incidence of post dural puncture headache (PDPH) after accidental dural puncture by a Tuohy needle is high [3]. We report a patient who developed a subdural hematoma (SDH) after ADP by epidural anesthesia without a remarkable headache.

## Case presentation

A 41-year-old parturient female, 2 gravida, 2 para, with a history of a previous cesarean delivery, was scheduled to undergo elective cesarean delivery. Past medical history revealed that she had been diagnosed as having an unruptured cerebral aneurysm with a diameter of 3 mm by magnetic resonance angiography. Her previous cesarean delivery was uneventful. The results of preoperative blood examination including platelet count, prothrombin time, and activated prothromboplastin time were normal. Epidural catheterization was attempted using an 18-gauge Tuohy needle (UNIVER, UNISYS, Saitama, Japan) through the Th12/L1 interspace. Because cerebrospinal fluid (CSF) leakage was identified during puncture of the epidural space, the epidural catheter was placed at the Th11/12 interspace after re-puncture of the epidural space. Spinal anesthesia was performed using a 25-gauge Quincke needle (UNISYS) at the L3/4 interspace. A bolus of isobaric 0.5% bupivacaine, 2.2 ml, was injected, and a dermatomal level of sensory block at the level of Th10 was confirmed by alcohol swab. The surgery was uneventful. Continuous epidural infusion of 0.25% levobupivacaine 4 ml/h was started after delivery using a continuous epidural infusion pump.

**Fig. 1:**
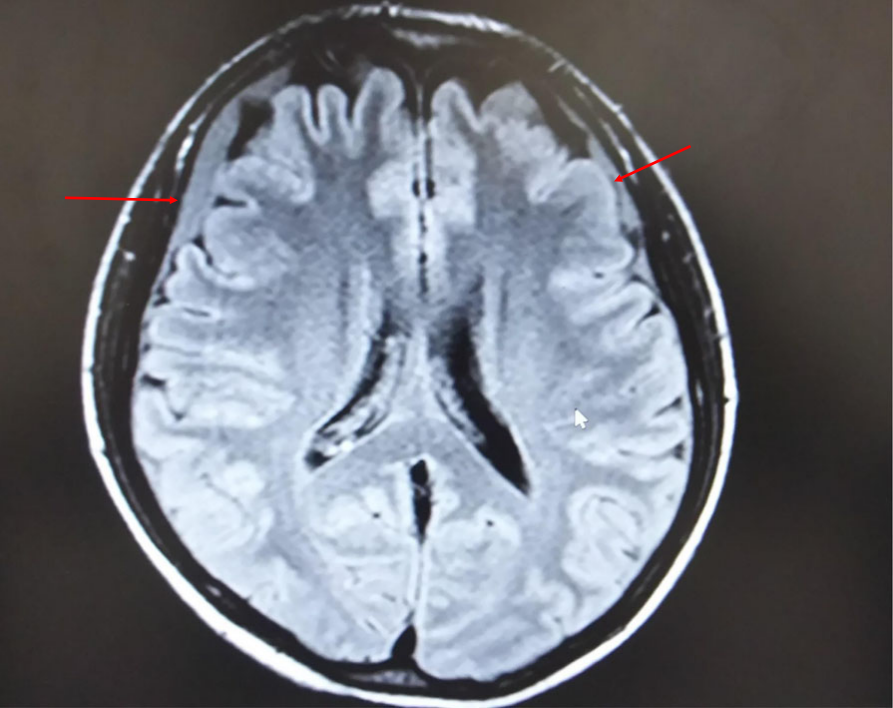
Magnetic resonance imaging performed 4 days after cesarean delivery. A bilateral subdural hematoma is shown (red arrow)

**Fig. 2:**
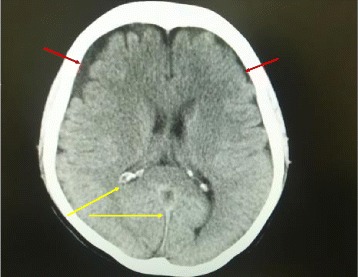
Computed tomogram obtained 9 days after the surgery. An increase in the size of the hematoma (red arrows) compared with that on the earlier MRI image is observed. A narrowed ventricle and midline shift (yellow arrows) are also observed, suggesting an increase in intracranial pressure

Her postoperative course was normal except for incisional pain. Loxoprofen 60 mg was administered as needed for incisional pain from the first postoperative day. On the second day after the surgery, the epidural catheter was removed and the patient complained of slight neck stiffness. Because the neck stiffness was slight, no treatment was administered. The patient could walk around the gynecological ward and no additional treatment was required except for administration of loxoprofen as needed. On the fourth postoperative day, administration of loxoprofen was discontinued because of improvement in incisional pain. Magnetic resonance imaging (MRI) was performed to ensure that her neck stiffness was not related to an intracranial problem on the fourth postoperative day; however, a bilateral subdural hematoma was observed (Fig. 1). After the neurosurgical consultation, it was decided that the patient would continue to be admitted in the hospital for observation without any surgical treatment. Absorption of the hematoma was expected. On the ninth postoperative day, she began to complain of headache and computed tomography (CT) was performed. The CT images showed an increase in the size of the hematoma compared with that on the earlier MRI image, as well as a narrowed ventricle and midline shift, indicating an increase in intracranial pressure (Fig. 2). After consultation with the neurosurgeon again, an epidural blood patch was carefully performed on the 10th postoperative day at the Th11/12 interspace, and 20 ml of autologous blood was injected. Surgical resection of the hematoma was scheduled as a back-up plan in case her headache became severe or neurological symptoms developed. During the epidural blood patch, the patient complained of slight back pain, but she had no neurological symptoms such as coma, headache, or nausea. After the procedure, the patient was carefully monitored for the development of neurological symptoms by the neurosurgeon. Her headache disappeared 1 day after the blood patch. A decrease in the size of the hematoma was confirmed on the CT images obtained on the 11th postoperative day, and the patient was discharged on the 18th postoperative day. The patient has not developed any problems after discharge.

We experienced a case of SDH in a patient who received combined spinal and epidural anesthesia after cesarean delivery. The patient complained of slight neck stiffness on the second postoperative day; however, her postoperative course seemed to be good and she could walk around the gynecological ward. Because the patient did not complain of headache, we did not consider the presence of an intracranial problem. In the present case, SDH was confirmed in a patient who did not have remarkable symptoms. The time course in the present case was similar to that in a patient reported by Macon et al. who did not present a remarkable headache before the development of SDH [6]. In most patients who developed SDH, a headache presented before the development of SDH [4, 7, 8]. The most important finding in the present case is that SDH developed in a patient who did not have a headache after ADP using a Tuohy needle. Several possible reasons can be considered for the absence of headache just after the surgery.

In the present case, ADP occurred. Generally, the incidence of PDPH after ADP by a Tuohy needle is higher than the incidence of PDPH after spinal anesthesia, and PDPH after ADP using an epidural needle is often severe [3, 7, 8]. Almost 70% of patients who received ADP using a Tuohy needle developed PDPH. In the present case, the development of PDPH was expected because CSF leakage was obtained through the epidural needle. In the present case, we placed an epidural catheter for postoperative pain management and continuous infusion of 0.25% levobupivacaine was administered. A study in which an epidural catheter was inserted into the intrathecal space after unintentional dural puncture indicated that the inflammatory reaction induced by the catheter sealed the hole in the dura mater and reduced the incidence and severity of headache [9]. They reported that epidural catheter placement may reduce the incidence of PDPH after accidental dural puncture [9]. In the present case, one possibility is that the magnitude of intracranial hypotension after the surgery was not severe because of the presence of the epidural catheter [10]. Also, the administration of loxoprofen for incisional pain may have influenced the severity of headache. However, continuous CSF leakage may have reduced cerebrospinal dynamics, which may have resulted in bleeding from the bridging vein. A case of CSDH following PDPH after spinal anesthesia using a 27 G spinal needle was reported [11], and a case of CSDH that developed 6 months after PDPH was also reported [12]. These cases and the present case may indicate that a small amount of continuous CSF leakage may induce the development of SDH.

Another possibility is that a SDH developed soon after the surgery by intracranial hypotension induced by ADP in our case. Some patients developed SDH after ADP during labor epidural analgesia relatively soon after the procedure [13, 14]. Leakage of CSF from a dural hole leads to reduced volume of CSF and results in decreases in intraspinal and intracranial pressure. The change in cerebrospinal dynamics induces caudal movement of the spinal cord and brain which results in stretching of the dura, cranial nerve, and bridging vein, which in turn causes headache and bleeding from the vein. In the present case, the patient complained of slight neck stiffness on the second postoperative day. It is speculated that bleeding occurred on the first or second day after the surgery, and the mass of the hematoma may have begun to compensate the intracranial hypotension caused by ADP.

It is widely believed that pregnancy increases the incidence of stroke because of changes in coagulopathy, abdominal compression by the bearing down effort, and susceptibility of the bridging vein to bleeding during pregnancy. The incidence of stroke after cesarean delivery is high [15]. Stocks et al. [16] reported a patient with cerebral vein thrombosis that was misdiagnosed as PDPH. Hashimoto et al. [17] reported that epidural blood patch induced impairment of consciousness in a patient with an intracranial problem. Again, in the present case, the patient complained of neck stiffness on the second postoperative day and MRI was performed on the fourth postoperative day. It may have been better to perform MRI or CT on the day that neck stiffness appeared. In the present case, headache related to ADP may have been masked by the analgesics administered for incisional pain or may have already been compensated by the SDH that developed earlier after the surgery. It is important to follow up slight symptoms such as neck stiffness. We performed epidural blood patch for treatment. In a case series reported by Zeidan et al. [8], many patients with a postdural puncture headache complicated by subdural hematoma following spinal or epidural anesthesia, especially patients with a neurological deficit or severe headache, were treated by surgical resection, whereas treatment by epidural blood patch was rare. In the present case, we decided to treat our patient with epidural blood patch because she had no neurological deficit. Two cases of subdural hematoma found after epidural blood patch were reported and one of the cases developed seizure immediately after the epidural blood patch [18, 19]. Even though sealing of the dura defect and restoration of CSF pressure are expected by epidural blood patch, there is a possibility of rebound intracranial hypertension and neurological deterioration. A case series reported by Hashizume et al. [20] indicated the possibility that SDH may enlarge or reoccur after epidural blood patch and may require hematoma evacuation [20]. Patients should be monitored carefully after epidural blood patch.

## Conclusion

We experienced a patient who developed a SDH without a remarkable headache. It may be better to perform imaging studies such as CT after ADP, even in patients who do not complain of headache.

## Abbreviations

CSDH: Chronic subdural hematoma
CSF: Cerebrospinal fluid
CT: Computed tomography
PDPH: Postdural puncture headache
SDH: Subdural hematoma
